# Individual factors and vection in younger and older adults: How sex, field dependence, personality, and visual attention do (or do not) affect illusory self-motion

**DOI:** 10.1177/20416695241270302

**Published:** 2024-08-12

**Authors:** Brandy Murovec, Julia Spaniol, Behrang Keshavarz

**Affiliations:** 7984 Toronto Metropolitan University, Toronto, Canada; KITE-Toronto Rehabilitation Institute, 7989 University Health Network, Toronto, Canada; 7984 Toronto Metropolitan University, Toronto, Canada; 7984 Toronto Metropolitan University, Toronto, Canada; KITE-Toronto Rehabilitation Institute, 7989 University Health Network, Toronto, Canada

**Keywords:** vection, self-motion, age, sex, personality, visual attention, field dependence

## Abstract

An important aspect to an immersive experience in Virtual Reality is vection, defined as the illusion of self-motion. Much of the literature to date has explored strategies to maximize vection through manipulations of the visual stimulus (e.g., increasing speed) or the experimental context (e.g., framing of the study instructions). However, the role of individual differences (e.g., age, biological sex) in vection susceptibility has received little attention. The objective of the current study was to investigate the influence of individual-difference factors on vection perception in younger and older adults. Forty-six younger adults (*M*_age _= 25.1) and 39 older adults (*M*_age _= 72.4) completed assessments of personality traits, field dependence, and visual attention prior to observing a moving visual stimulus aimed at inducing circular vection. Vection was measured using self-reports of onset latency, duration, and intensity. Results indicated that, in both age groups, females experienced longer-lasting vection compared to males. Additionally, the level of field dependence was related to vection intensity and duration in males but not in females. Variability in vection intensity was best explained by a mixture of biological, perceptual, cognitive, and personality variables. Taken together, these findings suggest that individual factors are important for understanding differences in vection susceptibility.

The experience of self-motion in the absence of any physical movement is a perceptual illusion known as vection (Dichgans & Brandt, 1978; Fischer & Kornmüller, 1930). Vection occurs, for instance, when a stationary observer is exposed to a large-scale moving visual stimulus producing a sensation of self-motion in the opposite direction (Palmisano et al., 2015). Experiences of vection are common to those who travel by train. For example, an individual sitting in a stationary train observing a moving train on an adjacent platform may interpret the motion of the adjacent train as movement of their own train (Kooijman et al., 2022a). It is also common to experience vection in highly immersive visual displays such as Virtual Reality (VR) applications when observing dynamic visual content (Hettinger et al., 2014). Traditionally, vection was regarded as a purely visual phenomenon, but more recent research demonstrated that vection is in fact a multisensory phenomenon, elicited and facilitated by other sensory modalities such as auditory and tactile stimulations (Keshavarz et al., 2014; Murovec et al., 2021; for review see Riecke et al., 2023).

Much of the scientific literature on vection has focused on investigating strategies to maximize the sensation of vection. In this vein, three domains have been identified (1) stimulus properties (i.e., bottom-up aspects), (2) expectations and goals (i.e., top-down aspects), and (3) individual-difference factors (i.e., characteristics of the participant). For example, with respect to bottom-up factors, it has been repeatedly demonstrated that increasing the size of the field-of-view or the speed of the moving stimuli increases vection (Allison et al., 1999; Murovec et al., 2021; Tamada & Seno, 2015). With respect to top-down factors, studies have shown that awareness that self-motion is possible and stimulus realism can impact vection (D’Amour et al., 2021; Palmisano & Chan, 2004; Riecke et al., 2006). Lastly, individual-difference variables have also been discussed as potential factors affecting the occurrence and the intensity of vection, including personality traits as well as cognitive, sensory, and perceptual characteristics (D’Amour et al., 2021; Palmisano et al., 2018; Väljamäe & Sell, 2014). However, empirical evidence of the role of individual differences in vection is limited. Thus, the goal of the present study was to systematically probe the role of individual differences, specifically focusing on the role of biological sex, age, personality traits, field dependence, and visual attention.

Gaining a better understanding of how individual characteristics predict vection is important for several reasons. Given that vection has been discussed as a critical factor in increasing realism of virtual environments and, as a result, improving the user's level of presence in the virtual world (Bonato et al., 2008; Heeter, 1992), it is possible that individuals who experience vection more easily may also have a more immersive VR experience. With VR advancing as a utility in a variety of practical domains such as health care (Perez-Marcos et al., 2018) and training (Weelden et al., 2021), ensuring a highly immersive experience is critical and may facilitate the success of these programs. As such, it is relevant to identify individual characteristics that are associated with vection to ensure an optimized VR experience for all users. Additionally, visually induced motion sickness (VIMS), a phenomenon that has been often linked to vection (Keshavarz & Golding, 2022), has been shown to be strongly influenced by individual-difference factors such as age or biological sex (Flanagan et al., 2005; Klosterhalfen et al., 2006). Thus, it is important to understand whether similar individual-difference factors may also affect vection, adding further to our understanding of the relationship between vection and VIMS, which remains unclear to date (Keshavarz et al., 2015).

## Biological Sex

The influence of biological sex (i.e., sex assigned at birth) on vection has yielded mixed evidence across the literature. Some studies have found that there are no meaningful differences between males and females (Clifton & Palmisano, 2020; Jones, 1998; Lee et al., 1997; Wei et al., 2017) while others have demonstrated increased vection in females compared to males (Darlington & Smith, 1998; Kennedy et al., 1996). For example, in D’Amour et al. (2017), female participants reported stronger vection intensity scores after watching a first person video of a bicycle ride on a large projection screen. Similarly, Klosterhalfen et al. (2008) noted reduced vection onset latencies in females during exposure to black and white optokinetic stimuli in a vection drum. Explanations for why vection might be increased in females include sex-related differences in reporting strategies, perceptual biases in spatial orientation (Witkin et al., 1954), as well as anatomical differences in the vestibular system (Tremblay & Elliott, 2007). Relatedly, research in the field of VIMS, a phenomenon commonly co-occurring with vection (see Keshavarz et al., 2015), has found similar results, with some studies reporting increased symptomology in females compared to males (Flanagan et al., 2005; Koslucher et al., 2015), whereas other studies could not find any sex-related differences (Stanney et al., 2020).

## Age

Given that vection perception relies on a number of cognitive and sensory processes that change over the life course (i.e., visual motion perception, multisensory integration, sensory functionality; de Dieuleveult et al., 2017; Iwasaki & Yamasoba, 2014; Lich & Bremmer, 2014; Spear, 1993), it stands to reason that vection susceptibility may also change across the lifespan. For instance, it has been demonstrated that children typically experience more intense and longer-lasting vection compared to adults (Shirai et al., 2012, 2014, 2018). With regard to older age, there is currently no clear consensus on the existence and nature of adult age differences in vection. In some studies, older adults experienced more vection than younger adults (Bury et al., 2020; Paige, 1994). For example, Murovec et al. (2022) exposed older and younger participants to a rotating stimulus containing visual, auditory, and/or tactile cues aimed to induce circular vection. In this study, older adults reported stronger and longer sensations of vection compared to younger adults. The authors speculated that age-related decline in vestibular function may cause an overreliance on visual information when forming the self-motion percept. On the other hand, Haibach et al. (2009) found opposite results, suggesting a reduced propensity to experience vection in older adults. In this study, three age groups of participants (18–20, 60–69, and 70–79) stood on a force plate while observing an oscillating “moving room.” Whereas postural instability was greatest in the oldest participants, vection intensity was highest in the younger-adult group. While natural age-related changes to sensory processing and integration likely affect vection perception, another factor to consider is the higher incidence rate of sensory diseases and dysfunctions that are more common in older adults. For example, vision disorders such as glaucoma and age-related macular degeneration are more common in older age and have both been suggested to influence vection perception (Brin et al., 2019; Fushiki et al., 1999; Tarita-Nistor et al., 2008).

## Field Dependence

Field dependence (FD), defined as the extent to which external or internal sensory cues affect the perception of body position (Bendall et al., 2016), varies across individuals. Those who are highly field-dependent rely more strongly on external (e.g., visual) cues, whereas low field-dependent individuals rely more strongly on internal (e.g., proprioceptive, vestibular) cues (Boccia et al., 2016). Given that vection has been thought of as a measure of visual dependency in visual-vestibular interactions (Kim et al., 2015), it stands to reason that those with high vection susceptibility may also be more field-dependent. Empirical findings in this regard are generally in support of this speculation (D’Amour et al., 2021; Harm et al., 1998; Rothacher et al., 2018); however, Keshavarz et al. (2017b) demonstrated that this effect might be contingent on the display type upon which the visual stimulus is presented. In their study, participants completed the computerized rod and frame (CRAF; Bagust et al., 2005) task to measure FD, and then presented a black and white striped optokinetic stimulus on four different displays: an immersive VR dome, an array of three monitors, a single monitor, and a projection screen. They found that vection intensity was increased in highly field-dependent individuals exclusively when the stimulus was observed in the large immersive VR dome. The authors speculated that increased vection was not seen in the other displays potentially because participants were able to use the edges of the screens as a reference frame, thus overruling any effect of field (in)dependence.

## Personality Traits

Very limited research to date has focused on exploring relationships between personality traits and vection. Since meaningful relationships between aspects of personality and cognitive processes (i.e., mental imagery; Mast et al., 2001; mental rotation; Ozer, 1987), everyday behaviours (Paunonen & Ashton, 2001), and even perceived velocity of object motion (Dureman et al., 1957) have been demonstrated in the past, a potential link between personality traits and vection seems possible. That said, we are aware of only two studies that have directly investigated the relationship between vection and personality (D’Amour et al., 2021; Seno et al., 2011b). In D’Amour et al., participants were assessed on depersonalization (i.e., feelings of detachment and dissociation from oneself), trait anxiety, and social desirability prior to observing an optokinetic stimulus aimed to induce circular vection. A significant positive correlation was found between depersonalization and vection intensity, suggesting that participants who report higher susceptibility to “out of body” experiences are more likely to experience stronger vection. Here, the authors discussed the possibility that vection and experiences of depersonalization are both mediated by shared vestibular pathways. In Seno et al., the authors assessed self-consciousness, narcissism, and the Big Five (i.e., extraversion, agreeableness, openness, conscientiousness, and neuroticism; John et al., 1991). The authors found a significant negative relationship between narcissism and vection intensity, suggesting that individuals higher in narcissism perceived weaker sensations of vection. People with narcissistic tendencies are thought to also be high in egocentricity, a state in which the self is only mildly influenced by information in the surrounding environment. Seno et al. argued that the high egocentricity of narcissistic individuals might be related to a reduction in FD, and, thus, reduced vection. Given that the research in this area is sparse, there is a need to further explore how vection may be related to other personality traits that have not yet been investigated. For example, investigating the relationship between trait suggestibility and vection might be an interesting avenue that has not yet been explored. It is possible that the extent to which an individual “buys in” to suggestive information (e.g., that vection is very likely to occur) may predict subsequent vection experiences.

## Visual Attention

A small area of literature examining vection under conditions of cognitive load has suggested that an individuals’ attentional capacity may also influence vection; however, the direction of this effect remains unclear. To the best of our knowledge, only three studies have been conducted in this domain, each yielding different results. For instance, Trutoiu et al. (2008) found that vection occurred faster in participants who viewed a realistic stimulus simulating movement through a tunnel while simultaneously completing a secondary task that required participants to press a button when a target was observed onscreen. In contrast, Seno et al. (2011a) demonstrated that vection intensity decreased when participants were instructed to count the number of appearances of the letter “X” (among other distracter letters on screen) while simultaneously viewing an optokinetic stimulus simulating upward/downward self-motion. Most recently, Kooijman et al. (2022b) showed that audio-visual vection was unaffected by the simultaneous completion of a visual discrimination reaction time task.

Other research in this domain has noted an influential role of selective attention on vection perception. In Kitazaki and Sato (2003), participants were presented with two opposing patterns of visual motion on the same display (red dots moving upward, green dots moving downward) and instructed to attend to only one pattern of motion. It was found that vection was perceived in the opposite direction of the non-attended motion. The authors speculated that the non-attended motion was regarded as the “attentional periphery” and vection is known to be moderated by background or peripheral motion (Dichgans & Brandt, 1978; Nakamura & Shimojo, 1998; Seya et al., 2014). In a related vein of research, it has been demonstrated that habitual action video game players who are known to have improved attentional control (Bavelier & Green, 2019; Föcker et al., 2018) may experience more vection in virtual environments (Pöhlmann et al., 2022).

Generally speaking, research on the impact of sex, personality, FD, and visual attention on vection is limited and has yielded inconsistent findings. More research is therefore needed in these areas to advance knowledge of individual differences in vection. Interestingly, most of the previously mentioned individual factors change as a function of age. For example, FD has been shown to increase (Agathos et al., 2015; Chan & Yan, 2018; Schwartz & Karp, 1967) whereas visual attention ability has been shown to decrease with age (Hommel et al., 2004). Aspects of personality traits are also known to change across the lifespan (Leszko et al., 2016). For instance, narcissism, a trait previously discussed to negatively correlate with vection (Seno et al., 2011b), is known to decease in older adulthood (Weidmann et al., 2023). These age-related changes, coupled with the sparse literature on individual factors affecting vection, highlight the need for further research in this domain to better determine the relative contributions of each individual factor on vection in participants of different age groups.

## The Current Study

The goal of the current study was to investigate individual factors and their contribution to vection perception in different age groups. Information on participants’ age, biological sex, and gender^1^ was collected followed by assessments for FD, visual attention (i.e., processing speed, selective and divided attention), and personality (i.e., the Big Five and suggestibility). Participants were then exposed to a moving stimulus aimed at inducing the subjective sensation of self-rotation (i.e., circular vection). We hypothesized, based on previous work, that highly field-dependent individuals would experience more vection compared to individuals with low field dependence (D’Amour et al., 2021; Harm et al., 1998; Rothacher et al., 2018). Additionally, we hypothesized that a relationship between vection and visual attention would be found, indicating that individuals with better performance would experience increased vection (Pöhlmann et al., 2022). Based on previous studies, we expected older adults to be more field-dependent (which might suggest increased vection) but also poorer in visual attention (which might suggest reduced vection); therefore, our hypotheses with regards to age were exploratory and non-directional. In terms of personality traits, we hypothesized that individuals who experienced increased vection might also be higher in suggestibility; however, our hypotheses with regard to the Big Five traits were exploratory given the lack of evidence in support of a relationship in previous literature (Seno et al., 2011b). Lastly, we predicted that there would be no sex differences in vection based on previous literature (Clifton & Palmisano, 2020; Jones, 1998; Lee et al., 1997; Wei et al., 2017).

## Methods

### Participants

Forty-eight younger adults and 46 older adults participated in this study. Of those, nine participants (2 younger, 7 older adults) indicated that they had experienced no vection throughout the entire experiment and were therefore removed from the data analysis. This group of non-responders was relatively balanced in terms of sex (4 females, 5 males) and FD (5 high FD, 4 low FD). This subgroup of participants scored similarly on the visual attention tests and personality assessments relative to the means of the larger sample (see Table 2). This resulted in a final sample size of *N *= 85, with 46 younger and 39 older adults (see Table 1 for sample characteristics). This sample size allowed for the detection of medium-large effects ( η _p_^2 ^= .08) with 80% power to detect between-subjects effects in a mixed analysis of variance (ANOVA). Participants were first pre-screened to confirm that they were within the age range of interest (18–35; younger adults, 65+; older adults) and healthy (i.e., no self-reported recent history of stroke, psychiatric, vestibular, or musculoskeletal disorders). Additionally, the Early Treatment of Diabetic Retinopathy Study (ETDRS) chart (Ferris et al., 1982) was used to ensure participants’ visual acuity was within normal range (20/30 or better). All participants provided written consent prior to the experiment. The study was approved by the Research Ethics Boards of the University Health Network and Toronto Metropolitan University and was designed in accordance with the Ethical Principles of the American Psychological Association. Participants were free to stop the experiment at any time without negative consequences; however, all participants completed the experiment. Participants were compensated with a $15/hr CDN gift card.

**Table 1.** Demographic characteristics of the study sample by age and sex.

	Units	Younger adults (*n* = 46)	Older adults (n = 39)
Age	*M* ± *SD*	25.1 ± 4.67	72.4 ± 4.33
Age range		19–34	65–81
Female/male	*n*	33/13	22/17

**Table 2.** Descriptive statistics for the individual-difference factors in younger and older adults.

	Units	Younger adults	Older adults	*p* value
*Personality*	*M ± SD (n)*			
Extraversion		9.17 ± 2.89 (46)	8.28 ± 2.96 (39)	.21
Agreeableness		10.28 ± 2.12 (46)	11.18 ± 2.04 (39)	.07
Conscientiousness		11.51 ± 2.33 (46)	11.64 ± 2.07 (39)	.78
Openness		10.54 ± 2.08 (46)	11.03 ± 1.87 (39)	.29
Neuroticism		8.87 ± 2.70 (46)	10.72 ± 2.43 (39)	.002**
Suggestibility		48.02 ± 10.50 (46)	36.72 ± 7.60 (39)	<.001***
*Visual attention thresholds*	*M ± SD (n)*			
Processing speed		16.7 ± 0 (46)	27.58 ± 14.60 (37)	<.001***
Divided attention		16.7 ± 0 (46)	75.9 ± 43.5 (37)	<.001***
Selective attention		77.21 ± 24.60 (46)	213.14 ± 78.80 (37)	<.001***
*Field dependence*	*M ± SD (n)*			
Avg absolute error(˚)		1.35 ± .94 (46)	4.92 ± 4.07 (39)	<.001***

*Note: p*-values adjusted using the Benjamin–Hochberg correction. Two older adults did not complete the UFoV, resulting in a sample of *n *= 37 for the visual attention thresholds.

### Study Design

To investigate the effects of individual-difference factors on vection, a 2 × 2 × 2 between-subjects design was chosen that included the factors age (younger adults, older adults), sex (male, female), and FD (high, low). Note that the results presented in this paper were part of a larger research project (Murovec et al., submitted), in which we manipulated the realism (intact, scrambled) and speed (faster, slower) of the visual stimulus. However, for the purpose of the present study, we focus on the condition that produced the highest level of vection (i.e., intact image moving at faster speed)^2^ to maximize variability in the dependent variable.

### Stimuli and Apparatus

**Figure 1.**
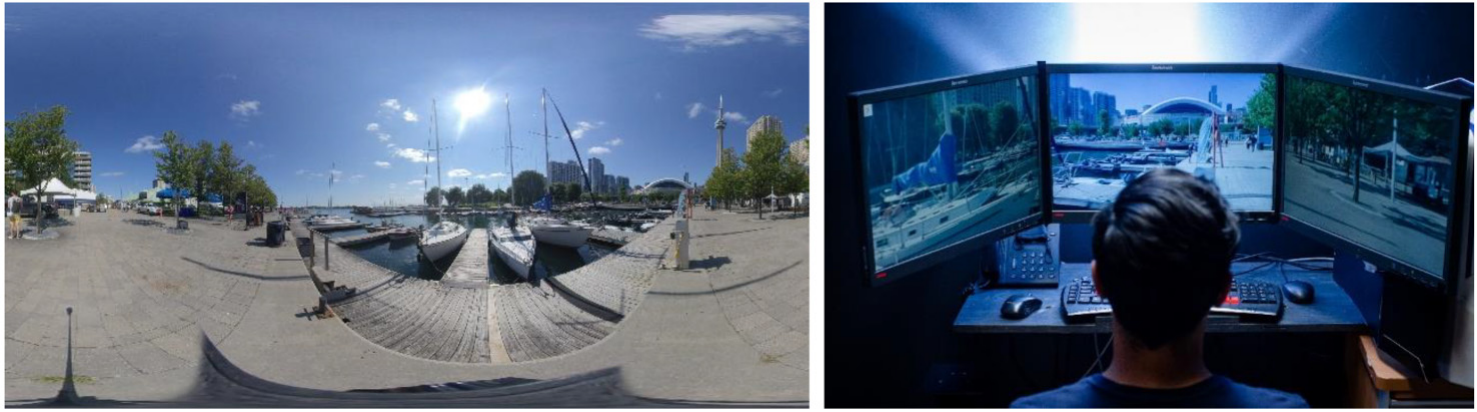
Screenshot of the 360° photo of Toronto Harbourfront used as the visual stimulus (left) and study setup including the array of three monitors showing the visual stimulus (right).

The visual stimulus consisted of a 360° panoramic photograph (Toronto Harbourfront) taken by an Insta 360 EVO camera with a 6080 × 3040 pixel resolution. The scene included several static objects (trees, benches) as well as iconic landmarks such as the CN Tower and the Rogers Centre (see Figure 1). The stimulus moved horizontally, either to the left or to the right, at a speed of 35˚/s. This motion was designed to induce the sensation of circular vection along the yaw axis, in the opposite direction of the visual motion. There was a total of six trials, each lasting 45 s and consisted of a static phase (2 s), an acceleration phase (3 s), constant motion (30 s), a deceleration phase (3 s), and a static phase (7 s). Between trials, the image disappeared, and the monitors turned black.

The stimuli were presented on an array of three 24-in. Lenovo (ThinkVision) monitors that were positioned adjacent to each other (approx. angle of 120° between monitors; see Figure 1). The refresh rate and display resolution were set to 60 Hz and 1,920 × 1,200 pixels, respectively. Participants sat in a rotatable chair with their head positioned 32 cm away from the centre screen, resulting in a field of view of approximately 228˚ horizontally and 48˚ vertically. The height of the chair was adjusted such that participants’ eye level aligned with the fixation cross in the centre of the middle screen. This setup has successfully induced vection in previous studies using different visual stimuli (D’Amour et al., 2021; Keshavarz et al., 2017a, 2017b).

### Dependent Measures

#### Vection Measures

We assessed three aspects of vection (Kooijman et al., 2023b): onset latency (i.e., the time it takes to first experience vection), duration (i.e., the total amount of time vection was experienced in a given trial), and intensity (i.e., the strength of the sensation). Onset latency and vection duration were measured using a button press system. Participants were instructed to press the left button of a wireless PC mouse as soon as vection began (onset) and hold the button pressed for the entire length of the sensation (duration). When vection stopped, participants were asked to release the button. Participants were able to indicate vection onset/offset as many times as needed during the trial. If participants experienced multiple onsets/offsets, the duration measure was captured by summing each time interval where the button was depressed. After each trial, vection intensity was measured using an 11-point scale ranging from 0 (no vection) to 10 (very strong vection). In addition to the vection measures, we also assessed any adverse effects related to VIMS using the Fast Motion Sickness Scale (Keshavarz & Hecht, 2011) after each trial and administered the Simulator Sickness Questionnaire (Kennedy et al., 1993) at the end of the experiment as a measure of participant well-being. Overall, VIMS ratings were low (average FMS score of *M *= 1.07) and therefore, will not be discussed further.

#### Personality Traits

To assess the Big Five (McCrae & Costa, 1996), the 10-item personality inventory (TIPI; Gosling et al., 2003) was used. Participants were instructed to rate the extent to which a pair of traits (e.g., extraverted, enthusiastic) applied to them on a 7-point Likert scale (1, disagree strongly; 7, agree strongly). A total score for each personality trait was computed by summing the values associated with the participant responses. Additionally, the Short Suggestibility Scale (SSS; Kotov et al., 2004) was used to measure general suggestibility. This scale contained 21 statements from five subscales of suggestibility (consumer suggestibility, persuadability, sensation contagion, physiological reactivity, and peer conformity) where participants were instructed to indicate the extent which they agreed on a 5-point Likert scale (1, not at all or very slightly; 5, a lot). A total score for general suggestibility was computed by summing the numerical values associated with the response options for each item, with larger scores indicating higher suggestibility.

#### Field Dependence

Prior to completing the experimental task, FD, visual attention, and personality traits were assessed. FD was measured by the CRAF test (Bagust et al., 2005), which involves changing the position of a rod (i.e., five linearly aligned dots) to be vertical within the surrounding square frame. Throughout the task, the frame was tilted clockwise, tilted counterclockwise, not tilted, or absent. FD was measured in terms of deviations in participants’ alignment with the true vertical, with larger deviations indicating higher FD. In our study, the CRAF was presented on a large projection screen (300 cm×196 cm) that participants viewed through a pair of clear ski goggles (no tint or lens strength) to occlude the peripheral visual field. Each of the four frame conditions was repeated four times. A final CRAF error score (averaged score of the absolute value of the difference in the tilted squares with the untilted square, in degrees) was calculated and used for the statistical analysis.

#### Visual Attention

Three aspects of visual attention were assessed using the useful field of view (UFoV) test (Ball et al., 1989): processing speed, divided attention, and selective attention. For processing speed, a two-alternative forced-choice task was used in which a single object (either a car or a truck) was shown on screen and participants indicated which type of object they saw. In the divided attention task, participants were again shown an object (either a car or a truck) in the middle of the screen, but another object (always a car) was simultaneously shown somewhere in the periphery. To complete this task, participants had to classify the object in the centre (car or truck), as well as indicate the location of the peripheral car. The selective attention task was identical to the divided attention task; however, the peripheral car was now being hidden among distractor items. For each subtest, presentation of the stimuli became quicker or slower based on a staircase procedure in order to determine processing thresholds (higher thresholds reflecting poorer performance).

### Procedure

Participants were informed that the purpose of the study was to investigate vection, which was described using the train analogy (Kooijman et al., 2023a). When participants had confirmed that they understood the concept of vection and had provided written consent, their visual acuity was assessed using the ETDRS chart. Older adults also completed the Montreal Cognitive Assessment (MoCA) to screen for cognitive impairment (score of 23 used as a cut off; Carson et al., 2018). All participants then completed the CRAF test to assess FD, followed by the UFoV test for visual attention, followed by the TIPI and SSS. Participants were then seated in the experimental chair where they first completed two practice trials to familiarize themselves with the vection task and procedure. Following the practice session, participants completed the six experimental trials, after which they were debriefed and compensated.

### Data Analysis

To examine the main effects and interactions of age, sex, and FD, a 2 (older, younger) × 2 (male, female) × 2 (high, low) ANOVA was conducted for each vection measure (onset latency, vection duration, vection intensity). The response variables were computed by averaging ratings across all trials, ignoring motion direction. Significant effects were followed up with Holm-corrected pairwise comparisons. To divide participants into higher and lower FD groups, a median split was performed on the average error in the CRAF for each age group to ensure equal representation of both age groups in each FD category (the medians for younger and older adults were 1.1 and 3.15, respectively). For trials that did not induce any vection, onset latency was set to 45 s (i.e., the maximum length of the trial; Palmisano et al., 2000, 2003). Additionally, associations between the vection measures and individual-difference factors were examined using correlations. Regression models with individual-difference factors as predictors were calculated for vection intensity, duration, and onset latency. Outlier detection using the 1.5*Interquartile range (IQR) guideline was applied to all numerical individual-difference variables (avg FD error, personality traits, visual attention thresholds) and winsorization with the 1.5*IQR thresholds was used. This approach was chosen to mitigate the influence of extreme values while preserving the overall distribution of the data. Given the Likert scale format of the personality questionnaires, Spearman correlations were computed between vection intensity and the Big Five traits as well as suggestibility, whereas Pearson correlations were computed for the remaining variables. All statistical analyses were performed using the statistical software R (R Core Team, 2021). Trials with missing data were removed from the analysis.

## Results

### Effects of Age, Sex, and FD on Vection

**Figure 2.**
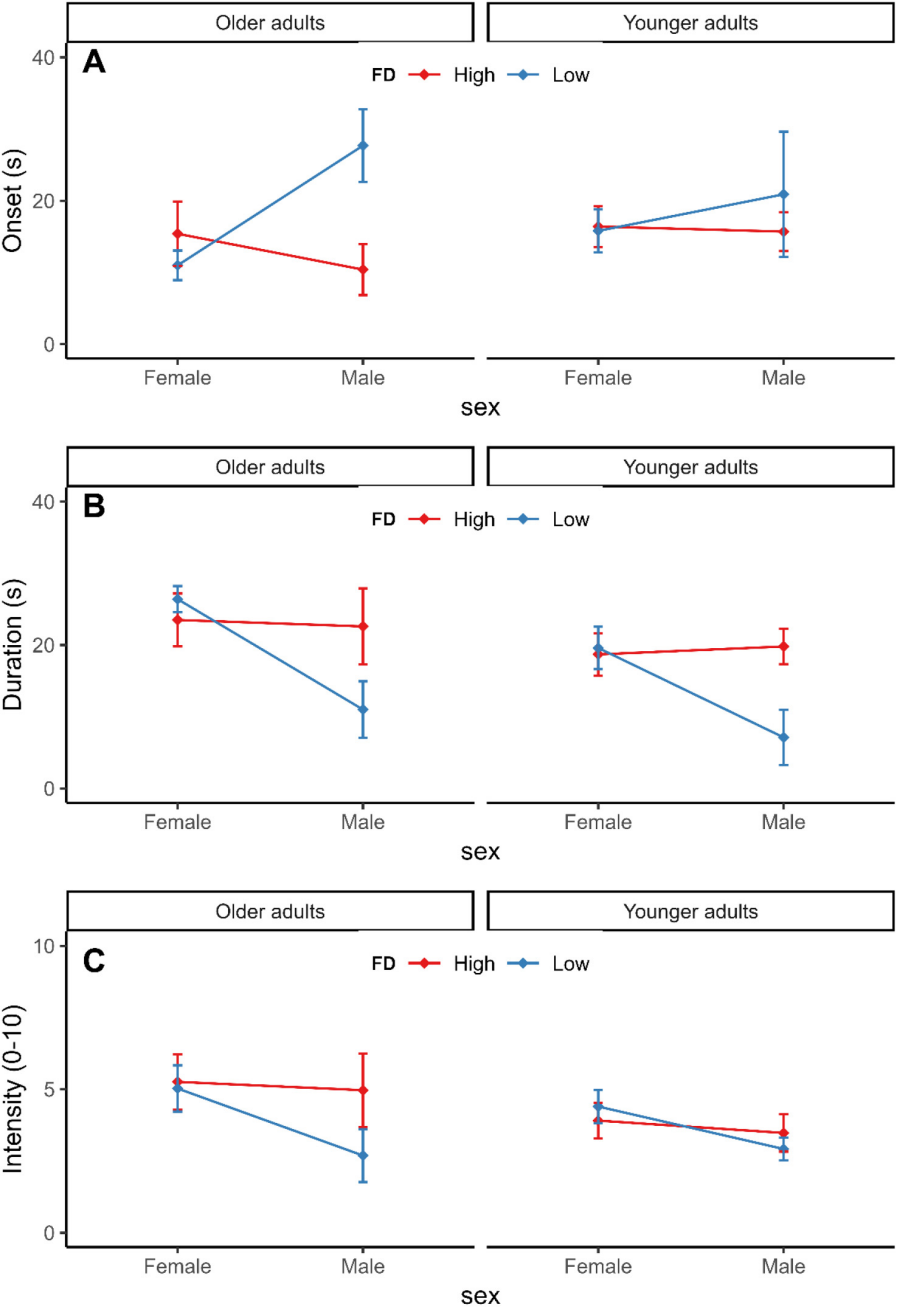
Average vection onset latency (A), vection duration (B), and vection intensity (C) ratings separated by sex, field dependence (FD), and age. Error bars represent +/– *SEM*.

Figure 2 shows the average onset latency (A), duration (B), and intensity (C) ratings as a function of sex, FD, and age group. For onset latency, there was a significant interaction of sex and FD, *F*(1,75) = 4.99, *p *< .05, η_p_^2^=.06. Post hoc tests indicated that vection occurred faster for higher versus lower field-dependent males, *t*(75) = −2.27, *p *< .05, but this effect was not observed for females, *t*(75) = .68, *p *> .05. For duration, a significant main effect of sex was found, *F*(1,75) = 6.27, *p *= .01, η_p_^2^=.08, with females reporting longer vection durations (*M* = 21.2, *SD* = 11.5) compared to males (*M* = 15.4, *SD* = 12). In addition, there was an interaction of sex and FD, *F*(1, 75) = 6.43, *p *= .01, η_p_^2^=.08, indicating that vection duration was significantly longer for higher versus lower field-dependent male participants, *t*(75) = 2.73, *p *< .01. There was no effect of FD in females *t*(75) = −.57, *p *> .05. For vection intensity, the ANOVA revealed no significant main effects or interactions.

### Predicting Vection Intensity From Individual-Difference Factors

Prior to probing correlations between vection intensity and individual-difference variables, we identified outlier values from 14 participants (7 for avg FD error, 5 for processing speed, 2 for selective attention, 1 for suggestibility, and 2 for conscientiousness) using the 1.5*IQR guideline. These datapoints were winsorized using 1.5*IQR as thresholds. The resulting descriptive statistics for the personality, visual attention, and FD variables are shown in Table 2, while correlations with vection intensity are shown in Table 3. Several small-to-moderate correlations were found, none of which were significant.

**Table 3.** Correlations between vection-dependent variables and individual-difference factors for younger and older adults.

	Younger adults			Older adults		
	Intensity	Duration	Onset	Intensity	Duration	Onset
Extraversion	−.01	.003	.05	−.10	−.22	.15
Openness	.23	.07	−.13	.08	−.19	.16
Conscientiousness	.04	.17	−.01	−.03	−.06	.04
Agreeableness	.02	−.20	.11	.27	.11	−.06
Neuroticism	−.23	.10	.06	.15	.11	−.09
Suggestibility	.17	−.17	.02	.11	.11	−.18
Processing speed	NA^3^	NA	NA	−.28	−.22	.22
Divided attention	NA	NA	NA	−.09	−.26	.21
Selective attention	.19	.26	−.26	−.14	−.22	.11
Field dependence error	.15	.08	.15	.20	.11	.20

*Note:* Spearman's *r* correlations were computed between vection and the personality factors, and Pearson's *r* was used for the remaining variables.

Linear regression models were calculated to estimate the amount of variance explained in vection intensity, vection duration, and onset latency by individual-difference factors. For vection intensity, assumptions of normality, linearity, and heteroscedasticity were met, but divided attention was found to be multicollinear (variance inflation factor (VIF) = 9.15). Inspection of a correlation matrix of the visual attention measures indicated a high degree of covariance between divided attention and the other measures (processing speed, *r *= .55; selective attention, *r *= .80). Thus, we decided to remove divided attention as a predictor, leaving the five personality scores, thresholds for processing speed and selective attention, the average absolute error in FD, age, and sex in the model. This model accounted for 17% of the variance in vection intensity, *F*(11, 71) = 2.56, *p *< .01 (Table 4). Among the predictors, sex, openness, processing speed, and FD were significant (*p*'s < .05). No significant results were found for the regression models with regards to vection duration (adjusted *R*^2 ^= 1%) and vection onset latency (adjusted *R*^2 ^= 2%), suggesting that the individual-difference factors did not explain any relevant amount of variance.

**Table 4.** Full regression model predicting vection intensity.

	B	*SE*	β	*t*	*p*	CI
Intercept	−1.74	3.0		−0.58	.58	[−7.73, 4.25]
Sex	1.53	.61	.27	2.50	.01**	[.31, 2.75]
Age	1.05	1.03	.20	1.02	.31	[−1.01, 3.12]
Extraversion	−.15	.10	−.16	−1.43	.15	[−.36, .06]
Agreeableness	.14	.14	.11	.96	.34	[−.15, .42]
Conscientiousness	.009	.13	.008	.076	.94	[−.24, .26]
Neuroticism	−0.07	.12	−.07	−0.60	.55	[−.30, .16]
Openness	.31	.15	.22	2.10	.04*	[.01, .60]
Suggestibility	.05	.03	.21	1.66	.10	[−.01, .11]
Processing speed	−.08	.03	−.31	−2.44	.02*	[−.13, −.01]
Selective attention	.0007	.005	.03	.15	.88	[−.01, .01]
Field dependence	.28	.11	.33	2.60	.01**	[.06, .49]

*Note: p*-values are two-tailed. df = 71.

We constructed another model containing the most relevant predictors accounting for an optimal amount of variance, using a forward stepwise regression approach. This model retained FD, sex, agreeableness, openness, and processing speed as predictors accounting for 18.6% (adjusted *R*^2^) of variance in vection intensity, *F*(5, 77) = 4.75, *p *< .001 (Table 5). Among these variables, FD, sex, and processing speed were significant (*p*'s < .05). The standardized regression coefficients indicated that FD had the strongest influence on vection intensity followed by processing speed where every 1 *SD* increase in these variables predicted a change in vection intensity by a factor of 0.36 and −0.25 *SD* units, respectively. For sex, intensity was predicted to increase by .23 *SD* units in female compared to male participants. The forward stepwise model was not significantly different from the full model, *F*(6, 77) = .80, *p *= .58.

**Table 5.** Forward stepwise regression predicting vection intensity.

	B	*SE*	β	*t*	*p*	CI
Intercept	−0.95	1.99		−.48	.64	[−4.92, 3.02]
FD	.30	.09	.35	3.35	.001	[.12, .48]
Processing speed	−.06	.03	−.25	−2.30	.02	[−.011, −.008]
Sex	1.30	.56	.23	2.32	.02	[.18, 2.42]
Openness	.24	.14	.18	1.74	.09	[−.03, .52]
Agreeableness	.20	.13	.15	1.51	.14	[−.06, .46]

*Note: p*-values are two-tailed. df = 77.

## Discussion

In the current study, we aimed to investigate the influence of various individual factors on vection in younger and older adults. Although we did not find any effects of age, sex was found to significantly impact vection, with females experiencing vection faster and longer than males. Additionally, vection was greater for males who were highly field-dependent compared to low. In the regression analysis, the best model (explaining more than 18% of variance) for vection intensity included the factors FD, sex, processing speed, openness, and agreeableness. Of these factors, FD (higher FD: larger errors in the CRAF), processing speed (faster processing speed: lower thresholds in the UFoV), and sex (females: higher intensity scores) were significant predictors. Openness and agreeableness were also included in the best model but did not reach statistical significance. We will discuss the roles of these factors in the following sections.

### Biological Sex, Field Dependence, and Vection

A small body of research investigating sex-related differences in vection has noted enhanced vection in females (D’Amour et al., 2017; Darlington & Smith, 1998; Kennedy et al., 1996; Klosterhalfen et al., 2008), consistent with the effect observed in the current study. In contrast, other studies did not find sex-related differences in vection; however, those studies were not necessarily designed to investigate biological sex as a factor contributing to vection, but rather balanced biological sex to control for potential effects, resulting in a smaller sample size and underpowered study designs for detecting sex-related differences in vection (Clifton & Palmisano, 2020; Lee et al., 1997). Explanations for this potential sex-related difference remain speculative at this point; one explanation could lie in differences in the weighting of sensory information during spatial orientation tasks. That is, it has been shown that males are more reliant on internal sensory cues (i.e., vestibular), whereas females tend to rely more heavily on external (contextual) cues (i.e., visual; Waber, 1977; Witkin et al., 1954). This finding has been repeatedly demonstrated in field dependence studies utilizing the rod and frame task, where females tended to perform less accurately and with higher variability than males in the presence of visual interference (Bogo et al., 1970; Groberg et al., 1969; Hyde et al., 1975). However, in the current study, we did not observe a difference in the FD task between male and female participants (*M*_femlaes _= 3.28, *M*_males _= 3.17, *p* = .90), questioning this line of argumentation. It has also been speculated that sex differences in vection perception may be a result of smaller otoliths (Sato et al., 1992) or increased postural instability (Wei et al., 2017) in females, but more evidence is needed to substantiate these claims.

Interestingly, an interaction between sex and FD was found, indicating that vection was sensitive to FD level in males but not females. While high and low FD females reported similar amounts of vection, a notable difference was observed between high versus low FD males. The finding of increased vection in high FD individuals is consistent with previous literature (D’Amour et al., 2021; Harm et al., 1998; Rothacher et al., 2018); however, it is surprising that females did not also follow this trend. The regression analysis largely corroborated these findings, where sex and FD were identified again as significant predictors of vection intensity.

### Age, Personality Traits, Visual Attention and Vection

The evidence for age effects in the context of vection is mixed. Relative to younger adults, older adults have been shown to experience increased vection in some studies (Murovec et al., 2022; Paige, 1994) and decreased vection in others (Haibach et al., 2009). The majority of the literature in this domain has shown no differences in younger versus older adults (Ishio et al., 2015; Keshavarz et al., 2017a; Murovec et al., submitted), consistent with the findings in the current study. Given the lack of consensus, it is likely that there are only small (if any) differences between older and younger adults, which may only be seen under certain experimental settings with ample power. For example, Murovec et al. (2022) used a highly immersive VR settings with multisensory stimulation presented on a large, dome-shaped laboratory, whereas the current study only used unimodal (i.e., visual) stimulation using a less-immersive study setup. Additionally, the current study induced circular vection about the yaw axis and found no age differences; however, this finding may not apply to different types/directions of vection (i.e., roll, pitch) and thus, should be further investigated.

In a related vein of research, age differences have been noted in static reorientation illusions (i.e., levitation illusions). The levitation illusion occurs when a stationary observer in a stationary environment perceives the direction of gravity to be different from the true direction of gravity (i.e., supine observers in a furnished tilted room). Howard et al. (2000) investigated vection (illusory self-tilt) as well as levitation illusions using a tumbling room in participants of various ages: children (*M* = 10 yrs), young adults (*M*_age _= 26 yrs), middle-aged adults (*M*_age _= 41 yrs), and older adults (*M*_age _= 72 yrs). While no age differences were reported in the vection conditions, it was found that the percentage of people experiencing levitation illusion increased with age. Given that the levitation illusion is thought to be driven by overreliance on visual information in forming a frame of reference, the authors speculated that older adults are especially dependent on visual information to compensate for loss in vestibular sensitivity. This finding suggests that age-related changes to sensory reliance can increase susceptibility to some (i.e., static) illusory experiences, but not others (i.e., dynamic). Further research should be conducted to better understand the conditions in which these age-related differences emerge.

The current study revealed small correlations between vection and individual-difference factors. For vection intensity, the most notable correlations (albeit not significant) were observed with openness (*r_s _*= .23) and neuroticism (*r_s _*= −.23) in younger adults and agreeableness (*r_s _= .*27) in older adults. For duration, the largest correlations were observed with selective attention (*r *= .26) in younger adults and divided attention (*r *= −.26) in older adults. For onset latency, the largest correlations were observed between selective attention (*r *= −.26) in younger adults and processing speed (*r *= .22) in older adults, although none of these associations reached significance.

To investigate the amount of variance explained in vection by individual factors, we aimed to construct regression models for each vection measure (intensity, duration, and onset latency); however, only the vection intensity models produced interesting results. In the full regression model, openness was a significant predictor suggesting that individuals who are more receptive to new ideas and experiences are more likely to experience stronger vection. However, in the stepwise regression model, openness and agreeableness were retained as influential variables but significance was only trending. These findings suggest that specific personality traits are linked to vection, but weakly. In terms of visual attention, processing speed was identified as a significant predictor of vection intensity. Specifically, those with lower thresholds (faster processing speed) were predicted to experience higher vection. This finding is consistent with a similar line of research investigating vection in action video game players, individuals who have been shown to have improved visual processing abilities (Bediou et al., 2018). In this study, action video game players reported experiencing strong vection illusions from rotating and expanding stimuli compared to non-gamers (Pöhlmann et al., 2022), suggesting enhanced processing speed may bolster vection. In a related area of research, Seno et al. (2011a, 2011b) investigated vection in a dual task paradigm where participants viewed an optokinetic stimulus while simultaneously completing a rapid serial visual presentation task. It was found that when cognitive load was high, vection was inhibited. Taken together with the current research, these findings suggest that a cognitive mechanism involving attentional resources may underlie vection perception, and thus individuals with heightened attentional abilities may be more likely to experience vection. Furthermore, this idea of an underlying mechanism may help to explain why cognitive manipulations (like expectation and plausibility) have been (at least partially) successful at modulating vection (D’Amour et al., 2021; Palmisano & Chan, 2004).

### Limitations and Future Directions

One limitation of the current study is that the visual stimulus/experimental set-up produced less strong vection than expected. The mean intensity rating was 4.14 out of a possible 10. Given that the effect of individual factors on vection is apparently small, using a more powerful vection stimulus may have helped to see the nature of these relationships more clearly. Another limitation was the use of the UFoV test to measure processing speed and divided attention in younger adults. As shown in the results, every younger adult scored perfectly in this portion of the test yielding no variability in the data. This lack of variability effectively eliminated the opportunity to examine the relationship between there variables and vection. In future, a different task measuring visual processing should be used, such as a modified Inspection Time task (Vickers et al., 1972) which utilizes similar methods (i.e., identifying a target object after a brief exposure) and has been shown to detect differences in younger adult samples (Ebaid & Crewther, 2019). Finally, it should be noted that the sex distribution was not equal. There were considerably more females than males, specifically in the younger adult age category (see Table 1). Statistically speaking, given that the number of observations per category directly influences the standard error, the likelihood of detecting sex differences naturally becomes greater. That said, the sex effect observed in the current study should be interpreted with caution and an equal sex ratio should be prioritized in future to remove any statistical bias from the analysis and confirm the validity of the sex effects.

Future research may consider examining other factors known to vary among individuals which may also have an influence on vection such as posturography and vestibular sensitivity. Previous literature in this domain has suggested that individuals with increased spontaneous postural instability experience more vection (Apthorp et al., 2014; Mursic et al., 2017). Additionally, individuals with increased vestibular sensitivity (lower thresholds) have been shown to experience vection quicker compared to individuals with lower sensitivity (Lepecq et al., 1999). However, these factors have yet to be tested in the context of vection with an older adult sample, and thus leave an area ripe for further investigation. Additionally, to further investigate the effect of FD on vection, future research could employ the rod and disk task to measure visual dependence more broadly. This task is similar to the CRAF where participants must align a rod to their perceived absolute vertical; however, some conditions in the rod and disk task have a moving dot background creating a visual illusion. Error between stationary and moving dot background conditions can be used as an index of otolith function and would be an interesting factor to investigate with vection. Lastly, the current study excluded participants who did not experience vection at all during the experiment. The goal of the present study was to identify factors which can predict vection characteristics such as intensity, which requires that participants must experience vection to some degree. However, identifying factors which characterize “non-responders” to vection is an interesting research question of its own and should be further investigated in future work.

### Conclusion

The current study investigated the influence of individual-difference factors on vection perception, a field of study which has received substantially less attention compared to stimulus properties or cognitive factors. Among these factors, we found that sex, FD, and processing speed were meaningful factors in understanding vection whereas age and personality traits were less so. This research suggests that vection can vary based on characteristics of the participant, and perhaps posits that individual-level information should be measured and reported on in vection literature to better contextualize the findings.

## Notes

1. There was no difference in responses between sex and gender for all participants; therefore, we only refer to one of these variables (i.e., sex) for the remainder of the paper.

2. Note that participant expectation was also manipulated in Murovec et al. but yielded no significant influence on vection and thus, was collapsed across the intact-fast condition used in the current study.

3. Correlations between the vection measures and processing speed and divided attention could not be computed for younger adults because there was no variability in the data. Every participant received a perfect score.

## References

[bibr1-20416695241270302] Agathos C. P. Bernardin D. Huchet D. Scherlen A.-C. Assaiante C. Isableu B. (2015). Sensorimotor and cognitive factors associated with the age-related increase of visual field dependence: A cross-sectional study. Age, 37, 67. 10.1007/s11357-015-9805-x 26122710 PMC4485658

[bibr2-20416695241270302] Allison R. S. Howard I. P. Zacher J. E. (1999). Effect of field size, head motion, and rotational velocity on roll vection and illusory self-tilt in a tumbling room. Perception, 28, 299–306. 10.1068/p2891 10615468

[bibr3-20416695241270302] Apthorp D. Nagle F. Palmisano S. (2014). Chaos in balance: Non-linear measures of postural control predict individual variations in visual illusions of motion. PLoS ONE, 9, e113897. 10.1371/journal.pone.0113897 PMC4252150 25462216

[bibr4-20416695241270302] Bagust J. Rix G. Hurst H. (2005). Use of a computer rod and frame (CRAF) test to assess errors in the perception of visual vertical in a clinical setting—A pilot study. Clinical Chiropractic, 8, 134–139. 10.1016/j.clch.2005.07.001

[bibr5-20416695241270302] Ball K. Beard B. Roenker D. Miller R. Griggs D. (1989). Age and visual search: Expanding the useful field of view. Journal of the Optical Society of America. A, Optics and Image Science, 5, 2210–2219. 10.1364/JOSAA.5.002210 3230491

[bibr6-20416695241270302] Bavelier D. Green C. S. (2019). Enhancing attentional control: Lessons from action video games. Neuron, 104, 147–163. 10.1016/j.neuron.2019.09.031 31600511

[bibr7-20416695241270302] Bediou B. Adams D. M. Mayer R. E. Tipton E. Green C. S. Bavelier D. (2018). Meta-analysis of action video game impact on perceptual, attentional, and cognitive skills. Psychological Bulletin, 144, 77–110. 10.1037/bul0000130 29172564

[bibr8-20416695241270302] Bendall R. C. A. Galpin A. Marrow L. P. Cassidy S. (2016). Cognitive style: Time to experiment. Frontiers in Psychology, 7, 1–4. https://www.frontiersin.org/articles/10.3389/fpsyg.2016.01786 10.3389/fpsyg.2016.01786 PMC5108774 27895616

[bibr9-20416695241270302] Boccia M. Piccardi L. Di Marco M. Pizzamiglio L. Guariglia C. (2016). Does field independence predict visuo-spatial abilities underpinning human navigation? Behavioural evidence. Experimental Brain Research, 234, 2799–2807. 10.1007/s00221-016-4682-9 27225254

[bibr10-20416695241270302] Bogo N. Winget C. Gleser G. C. (1970). Ego defenses and perceptual styles. Perceptual and Motor Skills, 30, 599–605. 10.2466/pms.1970.30.2.599 5454067

[bibr11-20416695241270302] Bonato F. Bubka A. Palmisano S. Phillip D. Moreno G. (2008). Vection change exacerbates simulator sickness in virtual environments. Presence: Teleoperators and Virtual Environments, 17, 283–292. 10.1162/pres.17.3.283

[bibr12-20416695241270302] Brin T. A. Tarita-Nistor L. González E. G. Trope G. E. Steinbach M. J. (2019). Vection responses in patients with early glaucoma. Journal of Glaucoma, 28, 68. 10.1097/IJG.0000000000001121 30461552

[bibr13-20416695241270302] Bury N.-A. Jenkin M. R. Allison R. S. Harris L. R. (2020). Perceiving jittering self-motion in a field of lollipops from ages 4 to 95. PLoS ONE, 15, e0241087. 10.1371/journal.pone.0241087 PMC7584255 33095827

[bibr14-20416695241270302] Carson N. Leach L. Murphy K. J. (2018). A re-examination of Montreal Cognitive Assessment (MoCA) cutoff scores. International Journal of Geriatric Psychiatry, 33, 379–388. 10.1002/gps.4756 28731508

[bibr15-20416695241270302] Chan J. S. Y. Yan J. H. (2018). Age-related changes in field dependence–independence and implications for geriatric rehabilitation: A review. Perceptual and Motor Skills, 125, 234–250. 10.1177/0031512518754422 29388513

[bibr16-20416695241270302] Clifton J. Palmisano S. (2020). Effects of steering locomotion and teleporting on cybersickness and presence in HMD-based virtual reality. Virtual Reality, 24, 453–468. 10.1007/s10055-019-00407-8

[bibr17-20416695241270302] D’Amour S. Bos J. Keshavarz B. (2017). The efficacy of airflow and seat vibration on reducing visually induced motion sickness. Experimental Brain Research, 235, 2811–2820. 10.1007/s00221-017-5009-1 28634889

[bibr18-20416695241270302] D’Amour S. Harris L. R. Berti S. Keshavarz B. (2021). The role of cognitive factors and personality traits in the perception of illusory self-motion (vection). Attention, Perception, & Psychophysics, 83, 1804–1817. 10.3758/s13414-020-02228-3 PMC8084801 33409903

[bibr19-20416695241270302] Darlington C. L. Smith P. F. (1998). Further evidence for gender differences in circularvection. Journal of Vestibular Research, 8, 151–153. 10.3233/VES-1998-8203 9547489

[bibr20-20416695241270302] de Dieuleveult A. L. Siemonsma P. C. van Erp J. B. F. Brouwer A.-M. (2017). Effects of aging in multisensory integration: A systematic review. Frontiers in Aging Neuroscience, 9, 1–14. 10.3389/fnagi.2017.00080 28400727 PMC5368230

[bibr21-20416695241270302] Dichgans J. Brandt T. (1978). Visual-vestibular interaction: Effects on self-motion perception and postural control. In Perception (pp. 755–804). Springer. 10.1007/978-3-642-46354-9_25

[bibr22-20416695241270302] Dureman I. Sälde H. Johansson G. (1957). Motion perception and personality II. Nordisk Psykologi, 9, 61–65. 10.1080/00291463.1957.11864014

[bibr23-20416695241270302] Ebaid D. Crewther S. G. (2019). Visual information processing in young and older adults. Frontiers in Aging Neuroscience, 11, 1–12. https://www.frontiersin.org/articles/10.3389/fnagi.2019.00116 10.3389/fnagi.2019.00116 PMC6532437 31156422

[bibr24-20416695241270302] Ferris F. L. Kassoff A. Bresnick G. H. Bailey I. (1982). New visual acuity charts for clinical research. American Journal of Ophthalmology, 94, 91–96. 10.1016/0002-9394(82)90197-0 7091289

[bibr25-20416695241270302] Fischer M. H. Kornmüller A. E. (1930). Optokinetisch ausgelöste Bewegungswahrnehmungen und optokinetischer Nystagmus [Perception of motion based on the optokinetic sense and optokinetic nystagmus]. Journal für Psychologie und Neurologie, 41, 273–308. 10.1007/978-3-642-91028-9_16

[bibr26-20416695241270302] Flanagan M. B. May J. G. Dobie T. G. (2005). Sex differences in tolerance to visually-induced motion sickness. Aviation, Space, and Environmental Medicine, 76, 642–646. 16018346

[bibr27-20416695241270302] Föcker J. Cole D. Beer A. L. Bavelier D. (2018). Neural bases of enhanced attentional control: Lessons from action video game players. Brain and Behavior, 8, e01019. 10.1002/brb3.1019 PMC6043695 29920981

[bibr28-20416695241270302] Fushiki H. Takata S. Nagaki Y. Watanabe Y. (1999). Circular vection in patients with age-related macular degeneration. Journal of Vestibular Research, 9, 287–291. 10.3233/VES-1999-9406 10472041

[bibr29-20416695241270302] Gosling S. D. Rentfrow P. J. Swann W. B. (2003). A very brief measure of the Big-Five personality domains. Journal of Research in Personality, 37, 504–528. 10.1016/S0092-6566(03)00046-1

[bibr30-20416695241270302] Groberg D. H. Dustman R. E. Beck E. C. (1969). The effect of body and head tilt in the perception of vertical: Comparison of body and head tilt with left and right handed, male and female subjects. Neuropsychologia, 7, 89–100. 10.1016/0028-3932(69)90048-7

[bibr31-20416695241270302] Haibach P. Slobounov S. Newell K. (2009). Egomotion and vection in young and elderly adults. Gerontology, 55, 637–643. 10.1159/000235816 19707011

[bibr32-20416695241270302] Harm D. L. Parker D. E. Reschke M. F. Skinner N. C. (1998). Relationship between selected orientation rest frame, circular vection and space motion sickness. Brain Research Bulletin, 47, 497–501. 10.1016/S0361-9230(98)00096-3 10052580

[bibr33-20416695241270302] Heeter C. (1992). Being there: The subjective experience of presence. Presence Teleoperators Virtual Environ, 1, 262–271. 10.1162/pres.1992.1.2.262

[bibr34-20416695241270302] Hettinger L. Schmidt-Daly T. Jones D. Keshavarz B. (2014). Illusory self-motion in virtual environments. In Handbook of virtual environments: Design, implementation, and applications (pp. 435–466). CRC Press. 10.1201/b17360-23

[bibr35-20416695241270302] Hommel B. Li K. Z. H. Li S.-C. (2004). Visual search across the life span. Developmental Psychology, 40, 545–558. 10.1037/0012-1649.40.4.545 15238042

[bibr36-20416695241270302] Howard I. P. Jenkin H. L. Hu G. (2000). Visually-induced reorientation illusions as a function of age. Aviation, Space, and Environmental Medicine, 71, A87–A91. 10993316

[bibr37-20416695241270302] Hyde J. S. Geiringer E. R. Yen W. M. (1975). On the empirical relation between spatial ability and sex differences in other aspects of cognitive performance. Multivariate Behavioral Research, 10, 289–309. 10.1207/s15327906mbr1003_3 26829631

[bibr38-20416695241270302] Ishio H. Yamakawa T. Sugiura A. Yoshikawa K. Kojima T. Terada S. Tanaka K. Miyao M. (2015). A study on within-subject factors for visually induced motion sickness by using 8 K display. In Antona M. Stephanidis C. (Eds.), Universal access in human-computer interaction. Access to interaction (pp. 196–204). Springer International Publishing. 10.1007/978-3-319-20681-3_18

[bibr39-20416695241270302] Iwasaki S. Yamasoba T. (2014). Dizziness and imbalance in the elderly: Age-related decline in the vestibular system. Aging and Disease, 6, 38–47. 10.14336/AD.2014.0128 25657851 PMC4306472

[bibr40-20416695241270302] John O. P. Donahue E. Kentle R. (1991). *Big Five Inventory*. 10.1037/t07550-000

[bibr41-20416695241270302] Jones S. A. (1998). Effects of restraint on vection and simulator sickness. ProQuest Information & Learning.

[bibr42-20416695241270302] Kennedy R. S. Hettinger L. J. Harm D. L. Ordy J. M. Dunlap W. P. (1996). Psychophysical scaling of circular vection (CV) produced by optokinetic (OKN) motion: Individual differences and effects of practice. Journal of Vestibular Research, 6, 331–341. 10.3233/VES-1996-6502 8887891

[bibr43-20416695241270302] Kennedy R. S. Lane N. E. Berbaum K. S. Lilienthal M. G. (1993). Simulator sickness questionnaire: An enhanced method for quantifying simulator sickness. The International Journal of Aviation Psychology, 3, 203–220. 10.1207/s15327108ijap0303_3

[bibr44-20416695241270302] Keshavarz B. Golding J. F. (2022). Motion sickness: Current concepts and management. Current Opinion in Neurology, 35, 107–112. 10.1097/WCO.0000000000001018 34839340

[bibr45-20416695241270302] Keshavarz B. Hecht H. (2011). Validating an efficient method to quantify motion sickness. Human Factors, 53, 415–426. 10.1177/0018720811403736 21901938

[bibr46-20416695241270302] Keshavarz B. Hettinger L. J. Vena D. Campos J. (2014). Combined effects of auditory and visual cues on the perception of vection. Experimental Brain Research, 232, 827–836. 10.1007/s00221-013-3793-9 24306440

[bibr47-20416695241270302] Keshavarz B. Novak A. C. Hettinger L. J. Stoffregen T. A. Campos J. L. (2017a). Passive restraint reduces visually induced motion sickness in older adults. Journal of Experimental Psychology: Applied, 23, 85–99. 10.1037/xap0000107 28150962

[bibr48-20416695241270302] Keshavarz B. Riecke B. E. Hettinger L. J. Campos J. (2015). Vection and visually induced motion sickness: How are they related? Frontiers in Psychology, 6, 1–11. 10.3389/fpsyg.2015.00472 PMC4403286 25941509

[bibr49-20416695241270302] Keshavarz B. Speck M. Haycock B. Berti S. (2017b). Effect of different display types on vection and its interaction with motion direction and field dependence. I-Perception, 8, 2041669517707768. 10.1177/2041669517707768 28515866 PMC5423592

[bibr50-20416695241270302] Kim J. Chung C. Y. L. Nakamura S. Palmisano S. Khuu S. K. (2015). The Oculus Rift: A cost-effective tool for studying visual-vestibular interactions in self-motion perception. Perception Science, 6, 248. 10.3389/fpsyg.2015.00248 PMC4358060 25821438

[bibr51-20416695241270302] Kitazaki M. Sato T. (2003). Attentional modulation of self-motion perception. Perception, 32, 475–484. 10.1068/p5037 12785485

[bibr52-20416695241270302] Klosterhalfen S. Muth E. R. Kellermann S. Meissner K. Enck P. (2008). Nausea induced by vection drum: Contributions of body position, visual pattern, and gender. Aviation, Space, and Environmental Medicine, 79, 384–389. 10.3357/ASEM.2187.2008 18457295

[bibr53-20416695241270302] Klosterhalfen S. Pan F. Kellermann S. Enck P. (2006). Gender and race as determinants of nausea induced by circular vection. Gender Medicine, 3, 236–242. 10.1016/s1550-8579(06)80211-1 17081956

[bibr54-20416695241270302] Kooijman L. Asadi H. Mohamed S. Nahavandi S. (2022a). A virtual reality study investigating the effect of cybersickness on the relationship between vection and presence across environments with varying levels of ecological relevance. 2022 15th International Conference on Human System Interaction (HSI), 1–8. 10.1109/HSI55341.2022.9869507

[bibr55-20416695241270302] Kooijman L. Asadi H. Mohamed S. Nahavandi S. (2022b). Does a secondary task inhibit vection in virtual reality? 2022 IEEE International Conference on Systems, Man, and Cybernetics (SMC), 1057–1064. 10.1109/SMC53654.2022.9945475

[bibr56-20416695241270302] Kooijman L. Asadi H. Mohamed S. Nahavandi S. (2023a). A virtual reality study investigating the train illusion. Royal Society Open Science, 10, 221622. 10.1098/rsos.221622 37063997 PMC10090874

[bibr57-20416695241270302] Kooijman L. Berti S. Asadi H. Nahavandi S. Keshavarz B. (2023b). Measuring vection: A review and critical evaluation of different methods for quantifying illusory self-motion. Behavior Research Methods, 393, 10.3758/513428-023-02148-8 PMC10991029 37369940

[bibr58-20416695241270302] Koslucher F. Haaland E. Malsch A. Webeler J. Stoffregen T. (2015). Sex differences in the incidence of motion sickness induced by linear visual oscillation. Aerospace Medicine and Human Performance, 86, 787–793. 10.3357/AMHP.4243.2015 26388085

[bibr59-20416695241270302] Kotov R. I. Bellman S. B. Watson D. B. (2004). *Multidimensional Iowa Suggestibility Scale (MISS) Brief Manual*. 16.

[bibr60-20416695241270302] Lee G. Yoo Y. Jones S. (1997). Investigation of driving performance, vection, postural sway, and simulator sickness in a fixed-based driving simulator. Computers & Industrial Engineering, 33, 533–536. 10.1016/S0360-8352(97)00186-1

[bibr61-20416695241270302] Lepecq J.-C. Giannopulu I. Mertz S. Baudonnière P.-M. (1999). Vestibular sensitivity and vection chronometry along the spinal axis in erect man. Perception, 28, 63–72. 10.1068/p2749 10627853

[bibr62-20416695241270302] Leszko M. Elleman L. G. Bastarache E. D. Graham E. K. Mroczek D. K. (2016). Future directions in the study of personality in adulthood and older age. Gerontology, 62, 210–215. 10.1159/000434720 26159881 PMC4706494

[bibr63-20416695241270302] Lich M. Bremmer F. (2014). Self-motion perception in the elderly. Frontiers in Human Neuroscience, 8, 681. 10.3389/fnhum.2014.00681 25309379 PMC4163979

[bibr64-20416695241270302] Mast F. W. Berthoz A. Kosslyn S. M. (2001). Mental imagery of visual motion modifies the perception of roll-vection stimulation. Perception, 30, 945–957. 10.1068/p3088 11578080

[bibr65-20416695241270302] McCrae R. Costa P. (1996). *The five factor model of personality: Theoretical perspective* .

[bibr66-20416695241270302] Murovec B. Spaniol J. Campos J. Keshavarz B. (2021). Multisensory effects on illusory self-motion (vection): The role of visual, auditory, and tactile cues. Multisensory Research, 34, 869–890. 10.1163/22134808-bja10058 34384047

[bibr67-20416695241270302] Murovec B. Spaniol J. Campos J. L. Keshavarz B. (2022). Enhanced vection in older adults: Evidence for age-related effects in multisensory vection experiences. Perception, 51, 698–714. 10.1177/03010066221113770 PMC9478596 35942780

[bibr68-20416695241270302] Murovec B. Spaniol J. Keshavarz B. (submitted). *Cognitive factors, age, and vection: The role of image realism and expectation on illusory self-motion perception in younger and older adults* .

[bibr69-20416695241270302] Mursic R. A. Riecke B. E. Apthorp D. Palmisano S. (2017). The Shepard–Risset glissando: Music that moves you. Experimental Brain Research, 235, 3111–3127. 10.1007/s00221-017-5033-1 28744623

[bibr70-20416695241270302] Nakamura S. Shimojo S. (1998). Stimulus size and eccentricity in visually induced perception of horizontally translational self-motion. Perceptual and Motor Skills, 87, 659–663. 10.2466/pms.1998.87.2.659 9842621

[bibr71-20416695241270302] Ozer D. J. (1987). Personality, intelligence, and spatial visualization: Correlates of mental rotations test performance. Journal of Personality and Social Psychology, 53, 129–134. 10.1037/0022-3514.53.1.129 3612485

[bibr72-20416695241270302] Paige G. (1994). Senescence of human visual-vestibular interactions: Smooth pursuit, optokinetic, and vestibular control of eye movements with aging. Experimental Brain Research, 98, 355–372. 10.1007/BF00228423 8050519

[bibr73-20416695241270302] Palmisano S. Allison R. Schira M. Barry R. (2015). Future challenges for vection research: Definitions, functional significance, measures, and neural bases. Frontiers In Psychology, 6, 10.3389/fpsyg.2015.00193 PMC4342884 25774143

[bibr74-20416695241270302] Palmisano S. Arcioni B. Stapley P. J. (2018). Predicting vection and visually induced motion sickness based on spontaneous postural activity. Experimental Brain Research, 236, 315–329. 10.1007/s00221-017-5130-1 29181555

[bibr75-20416695241270302] Palmisano S. Burke D. Allison R. S. (2003). Coherent perspective jitter induces visual illusions of self- motion. Perception, 32, 97–110. 10.1068/p3468 12613789

[bibr76-20416695241270302] Palmisano S. Chan A. Y. C. (2004). Jitter and size effects on vection are immune to experimental instructions and demands. Perception, 33, 987–1000. 10.1068/p5242 15521696

[bibr77-20416695241270302] Palmisano S. Gillam B. J. Blackburn S. G. (2000). Global-perspective jitter improves vection in central vision. Perception, 29, 57–67. 10.1068/p2990 10820591

[bibr78-20416695241270302] Paunonen S. Ashton M. (2001). Big five factors and facets and the prediction of behavior. Journal of Personality and Social Psychology, 81, 524–539. https://doi-org.ezproxy.lib.torontomu.ca/10.1037/0022-3514.81.3.524 11554651

[bibr79-20416695241270302] Perez-Marcos D. Bieler-Aeschlimann M. Serino A. (2018). Virtual reality as a vehicle to empower motor-cognitive neurorehabilitation. Frontiers in Psychology, 9, 10.3389/fpsyg.2018.02120 PMC6224455 30450069

[bibr80-20416695241270302] Pöhlmann K. M. T. O’Hare L. Dickinson P. Parke A. Föcker J. (2022). Action video game players do not differ in the perception of contrast-based motion illusions but experience more vection and less discomfort in a virtual environment compared to non-action video game players. Journal of Cognitive Enhancement, 6, 3–19. 10.1007/s41465-021-00215-6

[bibr81-20416695241270302] R Core Team (2021). *R foundation for statistical computing* [Computer software]. https://www.R-project.org/

[bibr82-20416695241270302] Riecke B. E. Murovec B. Campos J. L. Keshavarz B. (2023). Beyond the eye: Multisensory contributions to the sensation of illusory self-motion (vection). Multisensory Research, 1, 1–38. 10.1163/22134808-bja10112 37907066

[bibr83-20416695241270302] Riecke B. E. Schulte-Pelkum J. Avraamides M. N. Heyde M. V. D. Bülthoff H. H. (2006). Cognitive factors can influence self-motion perception (vection) in virtual reality. ACM Transactions on Applied Perception, 3, 194–216. 10.1145/1166087.1166091

[bibr84-20416695241270302] Rothacher Y. Nguyen A. Lenggenhager B. Kunz A. Brugger P. (2018). Visual capture of gait during redirected walking. Scientific Reports, 8, 10.1038/s41598-018-36035-6 PMC6299278 30568182

[bibr85-20416695241270302] Sato H. Sando I. Takahashi H. (1992). Computer-aided three-dimensional measurement of the human vestibular apparatus. Otolaryngology–Head and Neck Surgery, 107, 405–409. 10.1177/019459989210700311 1408226

[bibr86-20416695241270302] Schwartz D. W. Karp S. A. (1967). Field dependence in a geriatric population. Perceptual and Motor Skills, 24, 495–504. 10.2466/pms.1967.24.2.495 6040222

[bibr87-20416695241270302] Seno T. Ito H. Sunaga S. (2011a). Attentional load inhibits vection. Attention, Perception & Psychophysics, 73, 1467–1476. 10.3758/s13414-011-0129-3 21491162

[bibr88-20416695241270302] Seno T. Yamada Y. Ihaya K. (2011b). Narcissistic people cannot be moved easily by visual stimulation. Perception, 40, 1390–1392. 10.1068/p7062 22416597

[bibr89-20416695241270302] Seya Y. Tsuji T. Shinoda H. (2014). Effect of depth order on linear vection with optical flows. I-Perception, 5, 630–640. 10.1068/i0671 25926971 PMC4411986

[bibr90-20416695241270302] Shirai N. Endo S. Tanahashi S. Seno T. Imura T. (2018). Development of asymmetric vection for radial expansion or contraction motion: Comparison between school-age children and adults. I-Perception, 9, 2041669518761191. 10.1177/2041669518761191 29755720 PMC5937634

[bibr91-20416695241270302] Shirai N. Imura T. Tamura R. Seno T. (2014). Stronger vection in junior high school children than in adults. Frontiers in Psychology, 5, 1–6. https://www.frontiersin.org/articles/10.3389/fpsyg.2014.00563 10.3389/fpsyg.2014.00563 PMC4053762 24971067

[bibr92-20416695241270302] Shirai N. Seno T. Morohashi S. (2012). More rapid and stronger vection in elementary school children compared with adults. Perception, 41, 1399–1402. 10.1068/p7251 23513625

[bibr93-20416695241270302] Spear P. D. (1993). Neural bases of visual deficits during aging. Vision Research, 33, 2589–2609. 10.1016/0042-6989(93)90218-L 8296455

[bibr94-20416695241270302] Stanney K. M. Fidopiastis C. Foster L. (2020). Virtual reality is sexist: But it does not have to be. Frontiers in Robotics and AI, 7, 10.3389/frobt.2020.00004 PMC7805626 33501173

[bibr95-20416695241270302] Tamada Y. Seno T. (2015). Roles of size, position, and speed of stimulus in vection with stimuli projected on a ground surface. Aerospace Medicine and Human Performance, 86, 794–802. 10.3357/AMHP.4206.2015 26388086

[bibr96-20416695241270302] Tarita-Nistor L. González E. G. Markowitz S. N. Lillakas L. Steinbach M. J. (2008). Increased role of peripheral vision in self-induced motion in patients with age-related macular degeneration. Investigative Ophthalmology & Visual Science, 49, 3253–3258. 10.1167/iovs.07-1290 18390642

[bibr97-20416695241270302] Tremblay L. Elliott D. (2007). Sex differences in judging self-orientation: The morphological horizon and body pitch. BMC Neuroscience, 8, 10.1186/1471-2202-8-6 PMC1779793 17207289

[bibr98-20416695241270302] Trutoiu L. C. Streuber S. Mohler B. J. Schulte-Pelkum J. Bülthoff H. H. (2008). Tricking people into feeling like they are moving when they are not paying attention. *Proceedings of the 5th Symposium on Applied Perception in Graphics and Visualization*, 190. 10.1145/1394281.1394319

[bibr99-20416695241270302] Väljamäe A. Sell S. (2014). The influence of imagery vividness on cognitive and perceptual cues in circular auditorily-induced vection. Frontiers in Psychology, 5, 1–8. https://www.frontiersin.org/articles/10.3389/fpsyg.2014.01362 10.3389/fpsyg.2014.01362 PMC4253967 25520683

[bibr100-20416695241270302] Vickers D. Nettelbeck T. Willson R. (1972). Perceptual indices of performance: The measurement of “inspection time” and “noise” in the visual system. Perception, 1, 263–295. https://doi-org.ezproxy.lib.torontomu.ca/10.1068/p010263 4680931 10.1068/p010263

[bibr101-20416695241270302] Waber D. P. (1977). Biological substrates of field dependence: Implications of the sex difference. Psychological Bulletin, 84, 1076–1087. 10.1037/0033-2909.84.6.1076 928570

[bibr102-20416695241270302] Weelden E. V. Alimardani M. Wiltshire T. J. Louwerse M. M. (2021). Advancing the adoption of virtual reality and neurotechnology to improve flight training. 2021 IEEE 2nd International Conference on Human-Machine Systems (ICHMS), 1–4. 10.1109/ICHMS53169.2021.9582658

[bibr103-20416695241270302] Wei M. Luo J. Luo H. Song R. (2017). The effect of gender on vection perception and postural responses induced by immersive virtual rotation drum. 2017 8th International IEEE/EMBS Conference on Neural Engineering (NER), 473–476. 10.1109/NER.2017.8008392

[bibr104-20416695241270302] Weidmann R. Chopik W. J. Ackerman R. A. Allroggen M. Bianchi E. C. Brecheen C. Campbell W. K. Gerlach T. M. Geukes K. Grijalva E. Grossmann I. Hopwood C. J. Hutteman R. Konrath S. Küfner A. C. P. Leckelt M. Miller J. D. Penke L. Pincus A. L. Back M. D. (2023). Age and gender differences in narcissism: A comprehensive study across eight measures and over 250,000 participants. Journal of Personality and Social Psychology, 124, 1277–1298. 10.1037/pspp0000463 37184962 PMC10188200

[bibr105-20416695241270302] Witkin H. A. Lewis H. B. Hertzman M. Machover K. Meissner P. B. Wapner S. (1954). Personality through perception: An experimental and clinical study (pp. xxvi, 571). Harper.

